# Multi-spectral optical imaging of the spatiotemporal dynamics of ionospheric intermittent turbulence

**DOI:** 10.1038/s41598-018-28780-5

**Published:** 2018-07-12

**Authors:** Abraham C.-L. Chian, José R. Abalde, Rodrigo A. Miranda, Felix A. Borotto, David L. Hysell, Erico L. Rempel, David Ruffolo

**Affiliations:** 1 School of Mathematical Sciences, University of Adelaide, Adelaide, SA 5005 Australia; 2 Institute of Aeronautical Technology (ITA), São José dos Campos, SP 12228-900 Brazil; 3 National Institute for Space Research (INPE), P.O. Box 515, São José dos Campos, SP 12227-010 Brazil; 4 Department of Physics, Faculty of Science, Mahidol University, Bangkok, 10400 Thailand; 5 UnB-Gama Campus, and Plasma Physics Laboratory, Institute of Physics, University of Brasília (UnB), Brasília DF, 70910-900 Brazil; 6 Departamento de Física, Facultad de Ciencias Físicas y Matemáticas, Universidad de Concepción, Concepción, Chile; 7 Department of Earth and Atmospheric Sciences, Cornell University, Ithaca, New York 14850 USA

## Abstract

Equatorial plasma depletions have significant impact on radio wave propagation in the upper atmosphere, causing rapid fluctuations in the power of radio signals used in telecommunication and GPS navigation, thus playing a crucial role in space weather impacts. Complex structuring and self-organization of equatorial plasma depletions involving bifurcation, connection, disconnection and reconnection are the signatures of nonlinear evolution of interchange instability and secondary instabilities, responsible for the generation of coherent structures and turbulence in the ionosphere. The aims of this paper are three-fold: (1) to report the first optical imaging of reconnection of equatorial plasma depletions in the South Atlantic Magnetic Anomaly, (2) to investigate the optical imaging of equatorial ionospheric intermittent turbulence, and (3) to compare nonlinear characteristics of optical imaging of equatorial plasma depletions for two different altitudes at same times. We show that the degree of spatiotemporal complexity of ionospheric intermittent turbulence can be quantified by nonlinear studies of optical images, confirming the duality of amplitude-phase synchronization in multiscale interactions. By decomposing the analyses into North-South and East-West directions we show that the degree of non-Gaussianity, intermittency and multifractality is stronger in the North-South direction, confirming the anisotropic nature of the interchange instability. In particular, by using simultaneous observation of multi-spectral all-sky emissions from two different heights we show that the degree of non-Gaussianity and intermittency in the bottomside F-region ionosphere is stronger than the peak F-region ionosphere. Our results are confirmed by two sets of observations on the nights of 28 September 2002 and 9 November 2002.

## Introduction

## Nonlinear dynamics of equatorial plasma depletions

Equatorial plasma depletions (EPDs) related to density irregularities often occur in the nighttime ionosphere at low- and mid-latitudes. The first observation of EPDs was reported by Booker and Wells^1^ in 1938 when they discovered spread-F in the ionogram of backscattered signals of an ionosonde in Huancayo, Peru. Booker^2^ developed a theory of ionospheric turbulence to explain EPDs responsible for the scintillation of radio waves from stars. EPDs are produced by the generalized Rayleigh-Taylor instability, or more generally, interchange instability, which evolves nonlinearly to form EPDs along Earth’s magnetic flux tubes^3^. Farley *et al*.^4^ used the incoherent scatter observations of EPDs from the 50 MHz Jicamarca radar to constrain the theories for the origin of EPDs, and suggested that gradients of density and drift velocity might be possible sources of instability. Woodman and La Hoz^5^ suggested that the rising EPDs are the source of radar echoes detected by Farley *et al*.^4^. Recent satellite observations and numerical simulations indicated that small-scale magnetic field variations in the nighttime ionosphere can originate from EPDs^6^. The structures of EPDs are anisotropic as a result of the large divergence between ionospheric conductivities parallel and perpendicular to the geomagnetic field. If we consider the North-South (N-S) dimension to be a proxy for magnetic apex height, then it is evident why the structures of EPDs should be anisotropic. Due to the interchange instability, the EPD structures are stretched in the vertical (N-S) direction as flows are mainly along that direction.

The dynamical evolution of ionospheric EPDs leads to complex structuring and self-organization of depleted density structures such as bifurcation, connection, disconnection, and reconnection. Bifurcation of EPDs creates Y-shaped EPDs which have been observed by radars^7,8^, airglow images^9^, satellites^10^, and numerical simulations^11,12^. The equatorial ionosphere can be subject to an interchange instability after sunset which generates gradients in the background plasma density. This primary instability often leads to the formation of large-scale EPDs that can rise to high altitudes in the topside F-region. Associated with these large-scale EPDs are small-scale secondary instabilities that can develop on the walls of EPDs^8,10^. A linear eigenmode analysis of secondary instabilities forming on an ascending circular EPD indicates that a single mode could not give rise to bifurcation, whereas a superposition of two modes with comparable and opposite amplitudes would; bifurcation in radar plumes occurs when the linear growth rate of secondary instabilities is greatest^8^. Numerical simulations of Huba and Joyce^11^ showed that the initial bifurcation of EPDs can be triggered by a Rayleigh-Taylor instability when the top of the EPD flattens at the head as it rises through the ionosphere, which can undergo multiple bifurcations in the F-region.

Disconnection (or pinching) of EPDs occurs when the rising regions of a depleted density structure disconnect from the lower regions in their ascent, which has also been observed by radars^7,8^, satellites^7,13^, airglow images^14,15^, and numerical simulations^12^. Laakso *et al*.^13^ showed that a downward motion of the plasma within the EPDs can occur if the background westward zonal electric field and upward vertical neutral wind in the equatorial ionosphere are sufficiently strong. A simultaneous occurrence of the updrafting and downdrafting plasma flows in a single EPD structure may cause the disconnection of the lower part of the EPD from its upper part. Hysell^8^ showed that an EPD can narrow to the point where disconnection occurs by diffusive dissipation if a small electric field normal to the background density gradient with the proper sign is present.

Reconnection (or merging) of EPDs has been observed by airglow images^15–17^, numerical simulations^11,16,18^, and satellites^18,19^. Merging can occur in three different ways: (i) several highly tilted EPDs from adjacent areas form a reconnected EPD structure consisting of several strings^18^; (ii) reconnection of a branch of an EPD arising from secondary instabilities with an adjacent EPD;^16^ (iii) reconnection of a slowing leading EPD, due to the reduction of its zonal drift speed, with another trailing EPD^15^. Broad plasma depletions are wide and deep decreases of plasma density whose longitudinal width is over several hundred kilometres, associated with plasma density reductions of large magnitude, which have been observed by satellites^19^, numerical simulations^18^, incoherent scatter radars^20^, radio-tomography images^21^, and airglow images^15^. Broad plasma depletions can be formed either by the merging of EPDs^15,18,19^ or ionospheric uplift^20^.

## Multi-spectral optical imaging of double EPD reconnection in the South Atlantic Magnetic Anomaly on 28 September 2002

**Figure 1:**
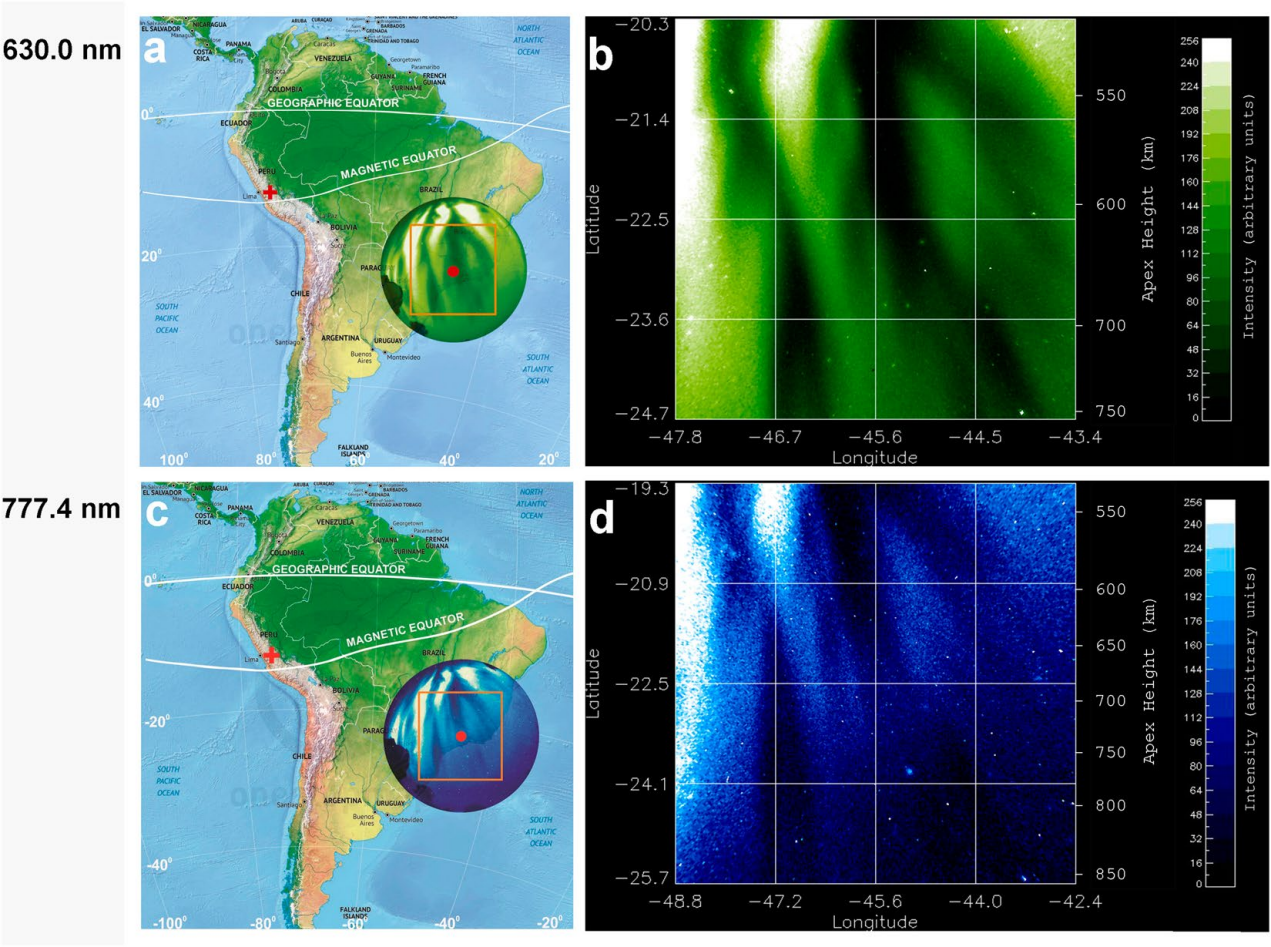
Multi-spectral optical imaging of equatorial plasma depletions on 28 September 2002. Map showing the projected circular field of view of the multi-spectral all-sky imager in the South Atlantic Magnetic Anomaly for: (**a**) the OI 630.0 nm emission peaked at an altitude ~280 km (bottomside F-region) and (**c**) the OI 777.4 nm emission peaked at an altitude ~330 km (peak F-region), observed on 28 September 2002 from Brazópolis, Brazil (marked by a red point). The orange boxes mark the areas of images to be investigated by quantitative analysis; the corresponding linearized grid images are divided into 16 cells covering an area of (**b**) 500 × 500 km^2^ and (**d**) 750 × 750 km^2^, respectively, indicated in latitude, longitude and magnetic apex height. The site of the first observation of equatorial plasma depletions by Booker and Wells in 1938 in Huancayo, Peru is marked by a red cross. The times of images in (**a**,**b**) (21:48:08 LT) and (**c**,**d**) (21:46:04 LT) correspond to Figs 2i and 4c and Figs 3i and 4f, respectively. The videos for this event can be found in Supplementary Videos S1 and S2. (**a**,**c** are modified from the Map of South America by onestopmap.com).

In optical images of airglow emissions, EPDs are observed as dark bands due to a decrease in emission intensity^3,9^. Fig. 1a,c shows near simultaneous all-sky images of OI 630.0 nm (21:48:08 LT) and OI 777.4 nm emissions (21:46:04 LT) observed on the night of 28 September 2002, indicating the field of view to 90° zenith angle of the multi-spectral all-sky (180°) imager located in Brazópolis (22.5°S, 45.6°W; dip latitude 17.5°S; altitude 1860 m), Brazil^14,22,23^. Top and right correspond to the north (N) and east (E), respectively. The back-illuminated charge-coupled device (CCD) (1024 × 1024 pixels) with high quantum efficiency of about 80% in the visible region was used for imaging. The raw images from the CCD camera were calibrated using the known star field to determine the pixel scale size and orientation of image data. The OI 630.0 nm and OI 777.4 nm emissions arise from the dissociative recombination of ${{\rm{O}}}_{2}^{+}$ ions and radiative recombination of O^+^ ions, respectively. Both emissions show large-scale EPDs aligned along the geomagnetic field lines, with a westward declination of about 20°, and with spatial resolution of the order of kilometers. The all-sky images are obtained with interference filters for the two emission lines, with a narrow bandwidth of only 2 nm to minimize the effects of viewing an optically thin target that adds effects at different altitudes. For filters with this narrow band, we mainly detect the emission lines of interest and the underlying continuum has not been removed. The peak OI 630.0 nm emission is from the bottomside F-region at an altitude ~280 km, while the peak OI 777.4 nm emission is from the peak F-region at an altitude ~330 km. These two F-region heights, each with a vertical width of about 50 km, are the source regions of the majority of the respective emissions.

Note that the raw images in Fig. 1a,c are distorted, curved, and compressed at the edges (low elevation angles) and inflated near the center or zenith (high elevation angles) due to the effect of the fish-eye lens. For any quantitative analysis, it is necessary to apply the linearization method to remove this effect^24,25^. Hence, we transform the box areas of raw images in Fig. 1a,c to linearized images of 500 × 500 km^2^ (Fig. 1b) and 750 × 750 km^2^ (Fig. 1d), respectively, with uniformly spaced geographic grids at an assumed emission altitude of 280 km (330 km) for the OI 630.0 nm (777.4 nm) emissions. The coordinates in Fig. 1b,d are given in latitude, longitude, and magnetic apex height projected to the magnetic equatorial plane^15,26–28^.

During the period of optical imaging on 28 September 2002, the geomagnetic activity was quiet. According to the IUGG (International Union of Geodesy and Geophysic) and NASA/OMNI data sets, the solar and geomagnetic indices were: the daily planetary index ΣKp (24-h sum of three-hourly index Kp) was 10-, the daily Ap index was 5, and the daily F10.7 solar flux index was 149.1. On the nights with no occurrence of EPDs, the ionospheric plasma density profile obtained by ionosounding was homogeneous, displaying a positive gradient with altitude in the bottomside F-region. On the night of 28 September 2002 when EPDs appeared on all-sky images, the ionospheric plasma density profile became inhomogeneous due to the presence of plasma density irregularities associated with spread-F^23^.

**Figure 2:**
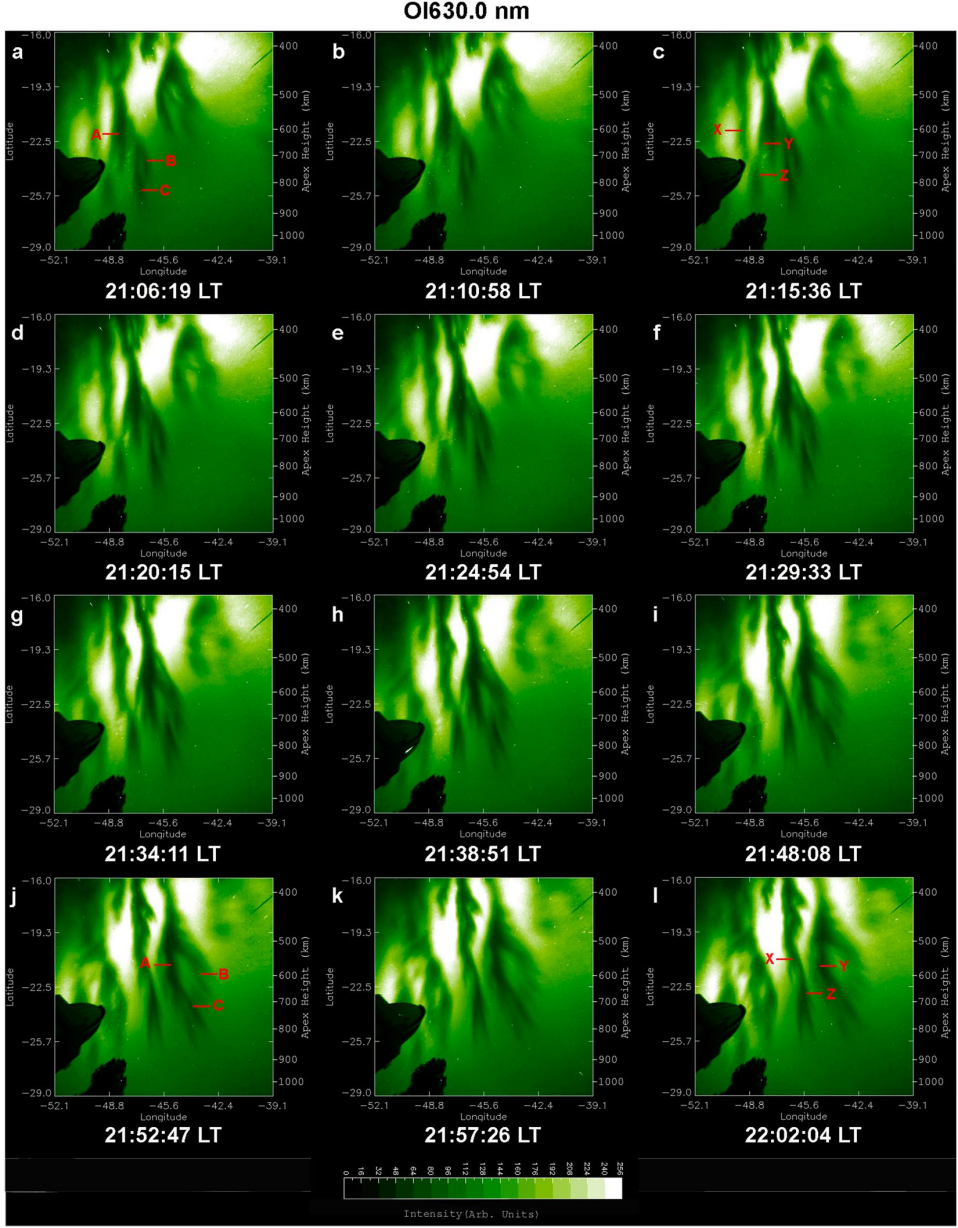
Overview of time sequence of all-sky images of the OI 630.0 nm emission observed on 28 September 2002. Each image has been linearized and projected to the magnetic equatorial plane onto an area of 1500 × 1500 km^2^ with uniformly spaced geographic grids at an assumed airglow emission height of 280 km. (A, B, C) denote EPD structures involved in the first EPD reconnection event to be described in detail in Fig. 4. The characterization of EPD dynamics for the first EPD reconnection event in terms of (A, B, C) for the images is given in Table 1. (X, Y, Z) denote EPD structures involved in the second EPD reconnection event to be described in detail in Fig. 5.

**Figure 3:**
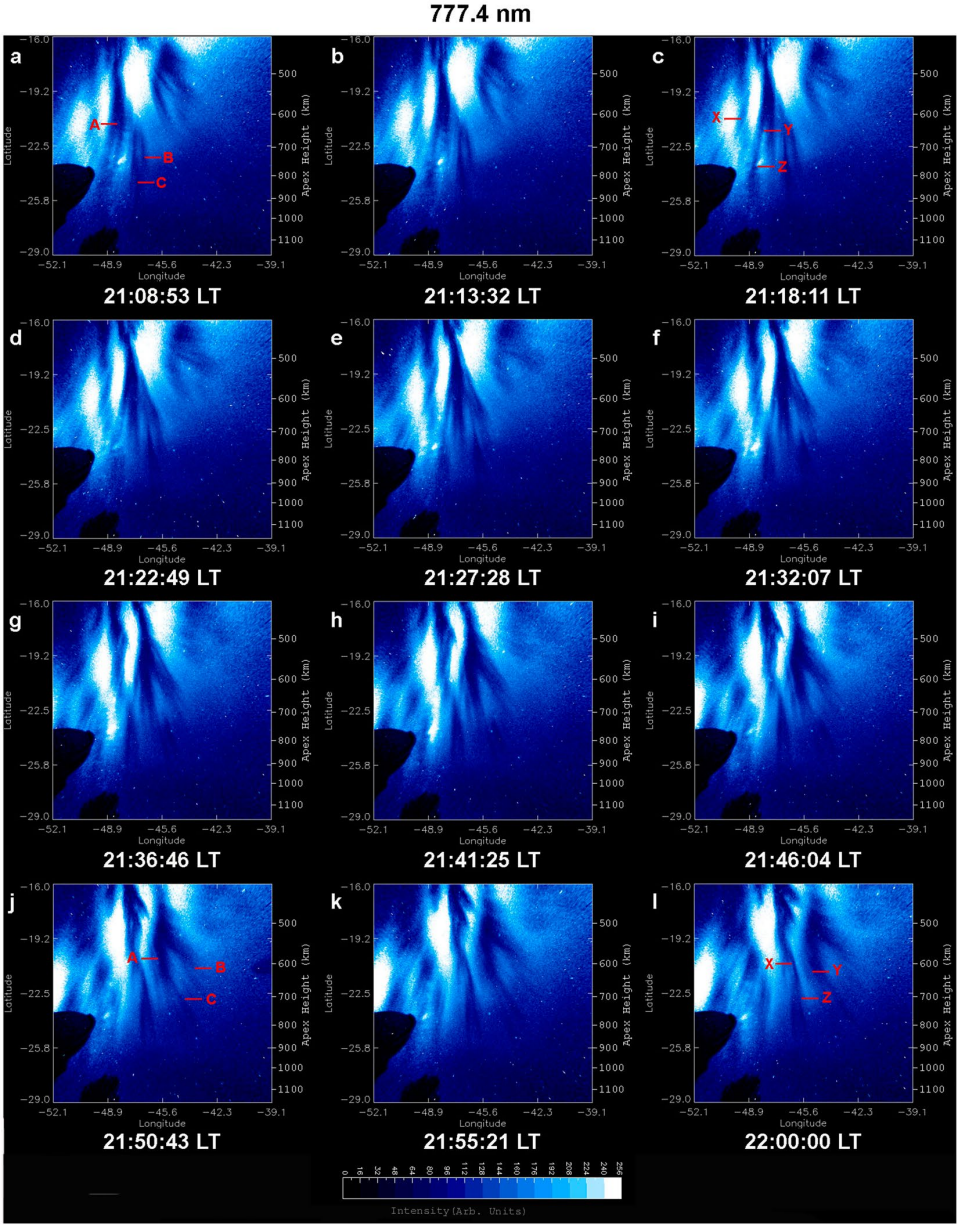
Overview of time sequence of all-sky images of the OI 777.4 nm emission observed on 28 September 2002. Each image has been linearized and projected to the magnetic equatorial plane onto an area of 1530 × 1530 km^2^ with uniformly spaced geographic grids at an assumed airglow emission height of 330 km. (A, B, C) denote EPD structures involved in the first EPD reconnection event to be described in detail in Fig. 4. The characterization of EPD dynamics for the first EPD reconnection event in terms of (A, B, C) for the images is given in Table 1. (X, Y, Z) denote EPD structures involved in the second EPD reconnection event to be described in detail in Fig. 5.

**Figure 4:**
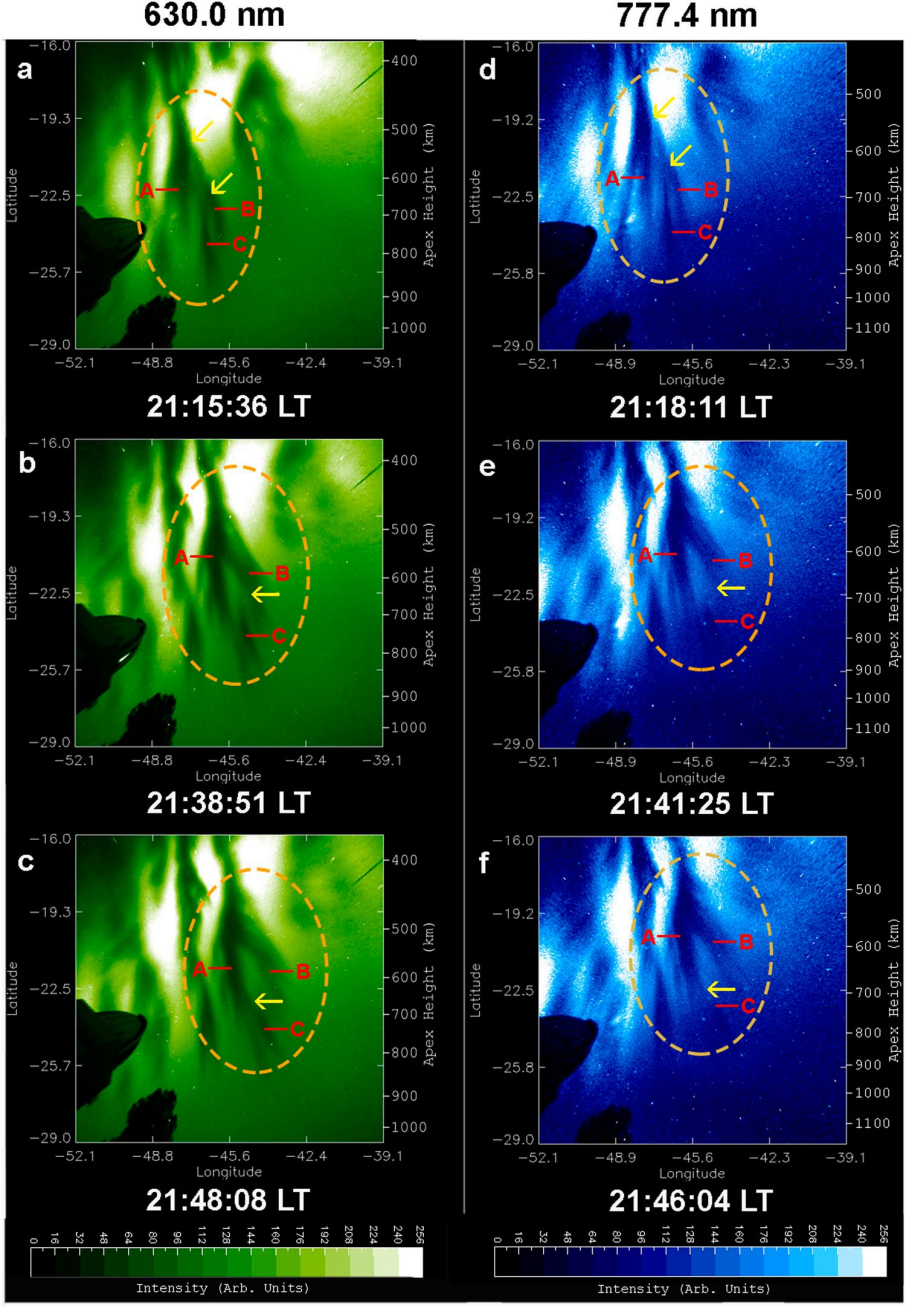
First EPD reconnection event on 28 September 2002. All-sky images of the OI 630.0 nm (left column) and OI 777.4 nm (right column) emissions observed on 28 September 2002. (A, B, C) denote EPD structures involved in the first EPD reconnection event. (**a**,**d**) A-B-C connected, the two arrows indicate the sites of bifurcation and connection of A and B-C and of B and C. (**b**,**e**) C disconnected from A-B, the arrow indicates the site of disconnection. (**c**,**f**) A-B-C reconnected, the arrow indicates the site of reconnection. The ellipses encircle the regions of dynamical interaction of EPD structures (A, B, C). The two Supplementary Videos provide a clearer view of EPD interactions.

**Figure 5:**
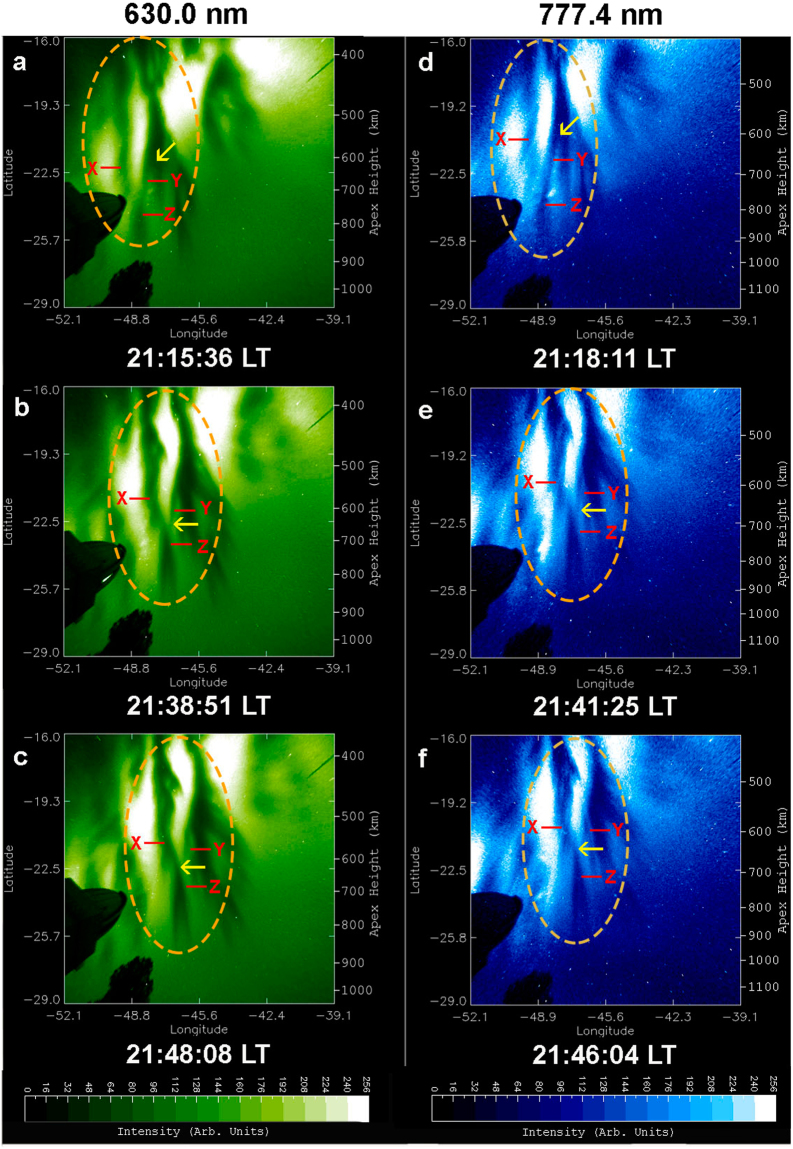
Second EPD reconnection event on 28 September 2002. All-sky images of the OI 630.0 nm (left column) and OI 777.4 nm (right column) emissions observed on 28 September 2002. (X, Y, Z) denote EPD structures involved in the second EPD reconnection event. (**a**,**d**) Y-Z connected, the arrow indicates the site of an EPD bifurcation. (**b**,**e**) Z disconnected from Y, the arrow indicates the site of disconnection. (**c**,**f**) X-Z reconnected, the arrow indicates the site of reconnection. The ellipses encircle the regions of dynamical interaction of EPD structures (X, Y, Z). The two Supplementary Videos provide a clearer view of EPD interactions.

An overview of optical imaging of the OI 630.0 nm and 777.4 nm emission on 28 September 2002 is given in Figs 2 and 3, which display a time sequence of 12 linearized images. To facilitate the visualization of EPD interactions the images in Figs 2 and 3 cover areas of 1500 × 1500 km^2^ and 1530 × 1530 km^2^, respectively, larger than the areas of Fig. 1b,d. These images exhibit the dynamical processes of multiple bifurcations, connection, disconnection, and reconnection of EPDs as classified in Table 1. In Figs 4 and 5 we describe in detail the first EPD reconnection event observed in the South Atlantic Magnetic Anomaly based on selected images of Figs 2 and 3.

**Table 1:** Time sequence of EPD dynamics for the first EPD reconnection event on 28 September 2002.

OI 630.0 nm (in Local Time)		OI 777.4 nm (in Local Time)	
21:06:19	A-B-C connected	21:08:53	A-B-C connected
21:10:58	A-B-C connected	21:13:32	A-B-C connected
21:15:36	A-B-C connected	21:18:11	A-B-C connected
21:20:15	A-B-C connected	21:22:49	A-B-C connected
21:24:54	A-B-C connected	21:27:28	A-B-C connected
21:29:33	A-B-C connected	21:32:07	A-B-C connected
21:34:11	C disconnected from A-B	21:36:46	C disconnected from A-B
21:38:51	C disconnected from A-B	21:41:25	C disconnected from A-B
21:48:08	A-B-C reconnected	21:46:04	A-B-C reconnected
21:52:47	A-B-C reconnected	21:50:43	A-B-C reconnected
21:57:26	A-B-C reconnected	21:55:21	A-B-C reconnected
22:02:04	A-B-C reconnected	22:00:00	A-B-C reconnected

A complex region of multiple EPDs is identified inside the ellipses of Fig. 4, where we can observe the dynamical phenomena of complex structuring and self-organization of the ionosphere by following the temporal evolution of three EPD structures: A, B and C. In Fig. 4a,d, A, B and C are connected at the two bifurcation sites indicated by two arrows. In Fig. 4b,e, C is disconnected^14^ from B at the disconnection site indicated by an arrow, while A remains connected to B. In Fig. 4c,f, C is reconnected to A at the reconnection site indicated by an arrow to form a merged elongated structure, the base of which is also connected to B. Table 1 and Fig. 4 show that the large-scale dynamics of EPD structures are similar at the two different altitudes at the same times. Another overview of optical imaging of this EPD reconnection event for smaller areas at the central regions of images similar to Fig. 1b,d, suitable for quantitative analysis, is given in Supplementary Fig. S1.

In addition to the aforementioned first EPD reconnection event, we have detected a second EPD reconnection event in the images of Figs 2 and 3, involving the interaction of three EPD structures denoted by X, Y and Z. Figure 5 covers the same regions of complex EPD interactions as Fig. 4 and provides the details of the second EPD reconnection event in the regions encircled by the ellipses. In Fig. 5a,d, Y and Z are connected forming an elongated EPD structure Y-Z, but X is not connected to Y-Z within the field-of-view of the all-sky imager; the arrow marks the site of EPD bifurcation. In Fig. 5b,e, Z becomes disconnected from Y at the disconnection site marked by an arrow. In Fig. 5c,f, X is reconnected to Z at the reconnection site marked by an arrow to form an elongated EPD structure X-Z. It is worth pointing out that the two EPD reconnection events shown in Figs 4c–f and 5c–f occur at same times in the same region of the ionosphere. Hence, we call this event “double EPD reconnection”. It is likely that multiple EPD reconnections are a common phenomenon in the evolution of Rayleigh-Taylor instability.

The three-dimensional numerical simulation of Huba *et al*.^16^ showed the development of multiple EPDs from initial perturbations near the equator and their ascension to high altitudes. Bifurcation and complex structuring involving the merger of two EPD structures were seen in this simulation. The detailed discussion of electrostatic reconnection of EPDs depicted in Figs 1 to 3 of Huba *et al*.^16^ is useful to interpret the two EPD reconnections we reported in Fig. 4c,f and in Fig. 5c,f. According to Huba *et al*.^16^, the EPD reconnection is a result of the connection of the electrostatic potential of an EPD with the electrostatic potential of an adjacent EPD which allows plasma flow from one EPD to another EPD through high-speed channels. This proposed reconnection mechanism provides a plausible physical interpretation for the occurrence of our observed EPD reconnections via the action of the electrostatic potential.

The first optical observation of EPD reconnection was reported by Huba *et al*.^16^ using an airglow imager of OI 630.0 nm emission in Tahiti on 5 July 2015. Initially two EPD structures were distinct and separated. Later, one EPD bifurcated and moved towards the other EPD until two structures merged at an altitude of around 550 km. Three different types of EPD reconnection processes were observed by Narayanan *et al*.^15^ using OI 630 nm imagers from Panhala, India, on 6 January 2008 and on 3 and 5 February 2008, and from Gandanki, India, on 23 March 2009, involving: (i) reconnection of various highly tilted EPDs from adjacent areas to form a merged structure; (ii) reconnection of a branch of an EPD resulting from secondary instabilities with a neighbouring EPD; (iii) reconnection of a slowing leading EPD, due to the reduction of its zonal drift speed, with another trailing EPD. EPD reconnection was observed by Xiong *et al*.^17^ using an OI 630.0 nm imager from Fuke, China, on the night of 23–24 September 2014. Initially, Xiong *et al*.^17^ observed bifurcation at a higher-latitude region of EPDs, and then at lower latitudes; the subbranches of EPD generated through bifurcations merge with each other to form a complicated mesh of plasma depletion regions. All previous observations of EPD reconnections were performed only by the OI 630.0 nm emission. The present paper is the first to report simultaneous multi-spectral observations of EPD reconnections at two heights. Since our observations and the observations of Huba *et al*.^16^ were carried out in the Southern Hemisphere, the EPDs first form at the top of the images and then ascend to higher magnetic apex heights toward the bottom of the images. In contrast, in the observations of Narayanan *et al*.^15^ and Xiong *et al*.^17^ carried out in the Northern Hemisphere, the EPDs first form at the bottom of the images and rise to higher magnetic apex heights toward the top of the images. Our multi-spectral observations confirm the occurrence of EPD bifurcation reported by Huba *et al*.^16^, Narayanan *et al*.^15^ and Xiong *et al*.^17^; as well as the occurrence of EPD disconnection seen in Fig. 3 of Narayanan *et al*.^15^ and Fig. 5 of Xiong *et al*.^17^. Both of our EPD reconnections shown in Figs 4 and 5 correspond to the third type of EPD reconnection process seen in Fig. 3 of Narayanan *et al*.^15^ and Fig. 5 of Xiong *et al*.^17^ involving first the disconnection of an EPD due to the change of its zonal drift speed, and its subsequent reconnection with an adjacent EPD to form an elongated structure.

## Nonlinear analysis of ionospheric intermittent turbulence on 28 September 2002

The rich dynamics of EPDs discussed in the previous section is the manifestation of nonlinear spatiotemporal evolution of primary and secondary instabilities of EPDs, leading to the formation of coherent structures and intermittent turbulence in the equatorial ionosphere. In this section, we show that nonlinear analysis of optical images of EPDs is capable of providing quantitative information of ionospheric intermittent turbulence.

**Figure 6:**
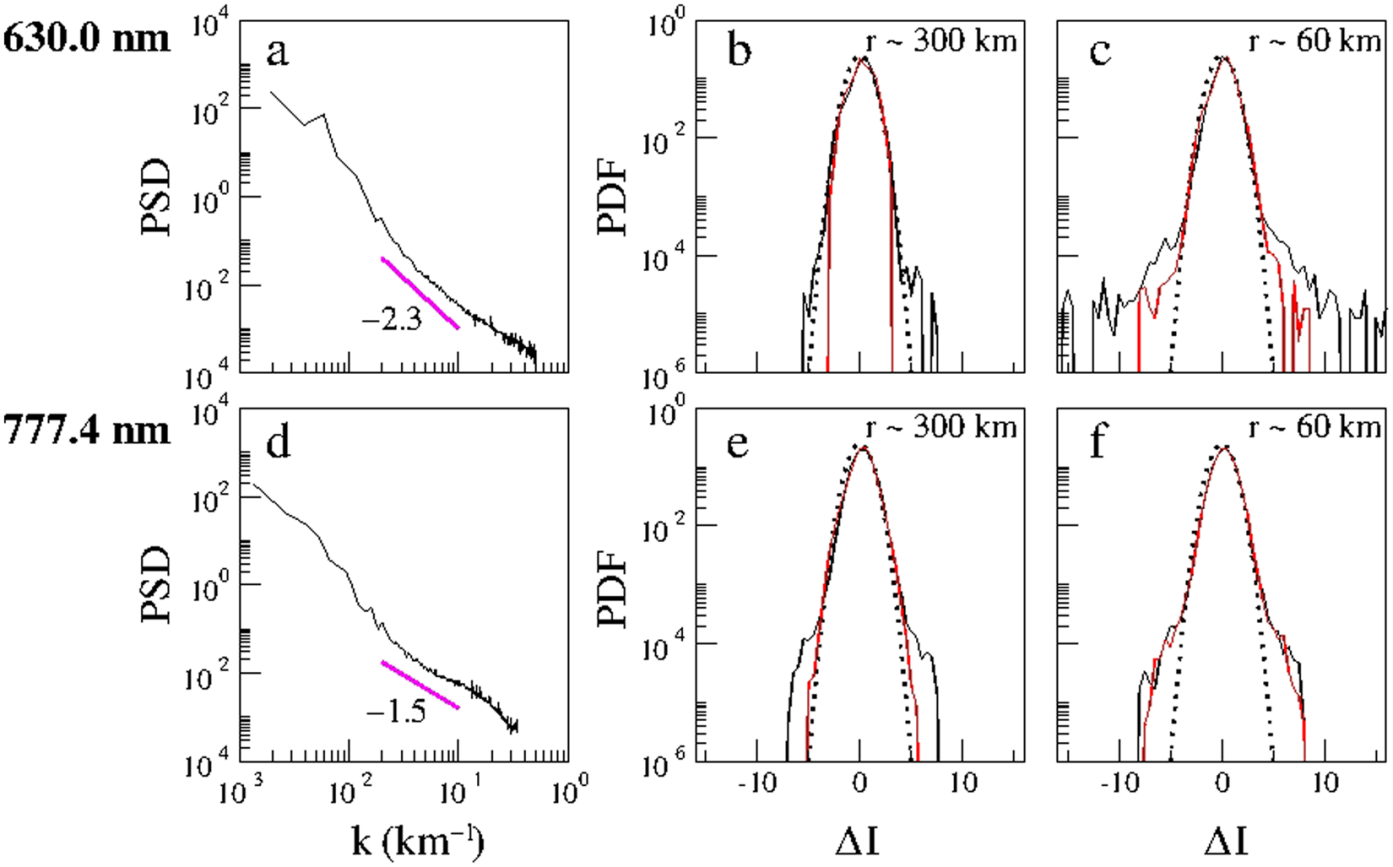
Power spectral density and probability density function of equatorial plasma depletions on 28 September 2002. Power spectral density (PSD) of the emission intensity *I* as a function of the wavenumber *k* of the: (**a**) OI 630.0 nm emission at 21:48:08 LT of Fig. 1b; (**d**) OI 777.4 nm emission at 21:46:04 LT of Fig. 1d. The straight line indicates the inertial subrange and the corresponding spectral index is given in (**a**,**d**). Probability density function (PDF) as a function of the normalized two-point differences of the emission intensity (Δ*I*) in the North-South direction (black) and East-West (red) direction of the: (**b**,**c**) OI 630.0-nm emission; (**e**,**f**) OI 777.4-nm emission for two spatial scales, *r* ~ 300 km and 60 km, superposed by a Gaussian PDF (dotted line).

Figure 6a,d shows the azimuthal average of the spatial power spectral density (PSD) of the emission intensity of OI 630.0 nm and OI 777.4 nm computed for the linearized images of Fig. 1b,d at 21:48:08 LT and 21:46:04 LT, respectively. Note that Fig. 1b,d is the same as Fig. S1c,f and its time is the same as Figs 2i, 3i, 4c,f and 5c,f. The straight line indicates the inertial subrange with *k* ~0.02 to 0.1 km^−1^ (i.e., for spatial scales *r*~300 to 60 km) as estimated by the compensated PSD method^29–31^, with a spectral index of −2.3 (−1.5). The validity of power spectra can be verified by computing the following relation between the power spectrum *P*(*k*) and the variance *σ*^2^ in the spatial domain, adapted from the corresponding relation in the temporal domain^32^

1 $${\int }_{-\infty }^{\infty }P(k)dk={\sigma }^{2},$$

where *P*(*k*) is given by Fig. 6a,d; the variance is normalized by the square of the mean emission intensity of the respective linearized image. For comparison, we also compute the normalized variance directly from the emission intensity *I* of the linearized images given by Fig. 1b,d. For the OI 630.0 nm emission, the normalized variance computed from both methods is 0.377. For the OI 777.4 nm emission, the normalized variance computed from the PSD is 0.407 and from the emission intensity is 0.409.

Figure 6b,c shows the probability density functions (PDFs) as a function of the normalized increment of the emission intensity Δ*I* = (*δI* − <*δI*>)/*σ*_*I*_, where *δI* denotes two-point differences of the emission intensity *I* for a given spatial scale *r*; the angular brackets denote the mean value of *δI*, and *σ*_*I*_ denotes the standard deviation of *δI* for two spatial scales, *r*~300 km and 60 km, respectively, of the emission intensity in the North-South (N-S, black) and East-West (E-W, red) directions of Fig. 1b superposed by a Gaussian PDF (dotted line). Figure 6e,f shows similar plots for the two spatial scales based on Fig. 1d. Note that the PDFs are closer to Gaussian distributions at large spatial scales, but become highly non-Gaussian at small spatial scales. At small spatial scales the shape of the PDF becomes leptokurtic, displaying fat tails in Fig. 6c,f, which are the signatures of nonlinear coherent (intermittent) structures responsible for large-amplitude fluctuations in the ionospheric turbulence. Figure 6c,f shows that the degree of intermittency and non-Gaussianity in the N-S direction is stronger than the E-W direction.

**Figure 7:**
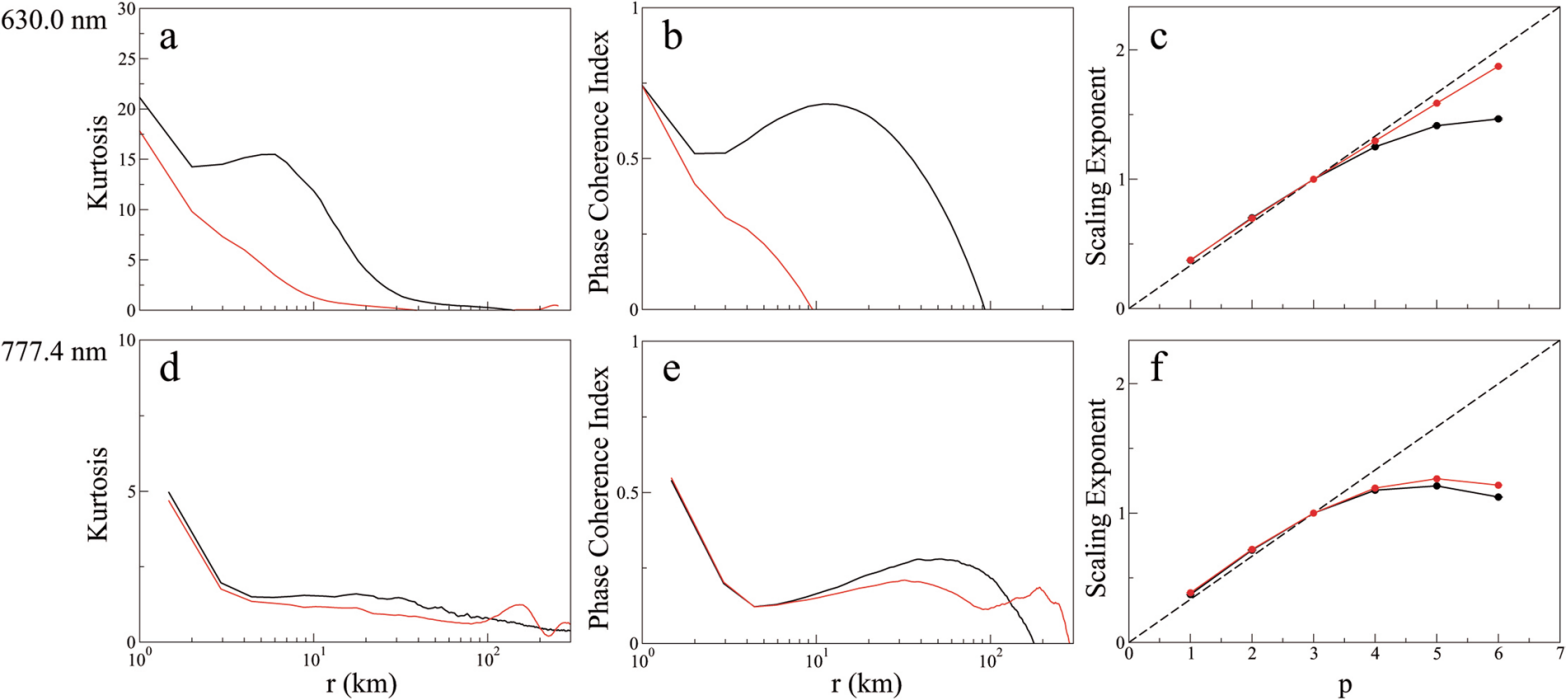
Nonlinear analysis of ionospheric intermittent turbulence on 28 September 2002. Kurtosis and phase coherence index as a function of the spatial scale *r*, and scaling exponents *ζ*(*p*) of the *p*th-order structure function for two-point differences of the emission intensity (*δI*) in the North-South direction (black) and East-West (red) direction of the: (**a**,**b**,**c**) OI 630.0 nm emission at 21:48:08 LT of Fig. 1b; (**d**,**e**,**f**) OI 777.4 nm emission at 21:46:04 LT of Fig. 1d. The Kolmogorov K41 self-similar scaling is indicated by a dashed line in (**c**,**f**).

The intermittent and non-Gaussian nature of the ionospheric turbulence can be quantified by computing the kurtosis and/or phase coherence index^29,31^. Kurtosis is a measure of amplitude synchronization in multiscale interactions, given by the normalized fourth-order moment

2 $$K(r)=\frac{1}{n}\sum _{i=1}^{n}{(\frac{\delta {I}_{i}-\langle \delta {I}_{i}\rangle }{{\sigma }_{I}})}^{4}-3,$$

where *δI*_*i*_ denotes two-point differences of the emission intensity *I* for a given spatial scale *r*; the angular brackets denote the mean values of *δI*_*i*_, and *σ*_*I*_ denotes the standard deviation of *δI*_*i*_. The variation of *K* as a function of *r* in the N-S (black) and E-W (red) directions for the images of Fig. 1b,d is plotted in Fig. 7a,d. For Gaussian fluctuations *K* = 0 for all scales, whereas for non-Gaussian or intermittent fluctuations *K* > 0. Figure 7a,d shows that *K* increases as the scale decreases, demonstrating that the ionospheric turbulence is intermittent and non-Gaussian over a broad range of spatial scales. In particular, they show that the degree of intermittency and non-Gaussianity in the N-S direction is stronger than the E-W direction. The difference between the two directions at the bottomside F-region is greater than the peak F-region.

Phase synchronization in multiscale interactions can be quantified by computing the phase coherence index^29^

3 $${C}_{\varphi }(r)=\frac{{S}_{PRS}(r)-{S}_{ORG}(r)}{{S}_{PRS}(r)-{S}_{PCS}(r)},$$

where

4 $${S}_{j}(r)=\sum _{i=1}^{n}|\delta {I}_{i}|,$$

with *j* = *ORG*, *PRS*, *PCS*. This index measures the degree of phase coherence in an original data set (*ORG*) by comparing it with two surrogate data sets constructed from the original data set: a phase-randomized surrogate (*PRS*) in which the phases of the Fourier modes are set fully random, and a phase-correlated surrogate (*PCS*) in which the phases of the Fourier modes are set fully equal. The power spectra of three data sets *ORG*, *PRS* and *PCS* are kept the same, but their phase spectra are different. C_*ϕ*_(*r*) = 0 implies that the phases of the fluctuations of the original data set are completely random, whereas C_*ϕ*_(*r*) = 1 implies that the phases are fully correlated. Figure 7b,e shows the variation of C_*ϕ*_ as a function of *r* in the N-S (black) and E-W (red) directions for the image of Fig. 1b,d. The behaviour of C_*ϕ*_ in Fig. 7b,e follows that of kurtosis in Fig. 7a,d, which is a demonstration of the duality of amplitude-phase synchronization^33^ in multiscale interactions in ionospheric intermittent turbulence. Note that we have removed the pixels with saturated values of the emission intensity from the linearized images before computing kurtosis and C_*ϕ*_(*r*).

The multifractal nature of ionospheric intermittent turbulence can be determined by computing the scaling exponent *ζ*(*p*) of the *p*th-order structure function. We apply the extended self-similarity technique^29,31^ to refine the calculation of the scaling exponent for the inertial subrange *k* ~0.02 to 0.1 km^−1^, so that the *p*th-order structure function ${S}_{p}(r) \sim {[{S}_{3}(r)]}^{\zeta (p)}$. Figure 7c,f shows *ζ*(*p*) decomposed in N-S (black) and E-W (red) directions for the images of Fig. 1b,d, respectively. The dashed line denotes the Kolmogorov K41 self-similarity scaling, *ζ*(*p*) = *p*/3. The computed scaling exponents show significant departures from self-similarity at higher moments, proving the multifractal nature of ionospheric intermittent turbulence as well as a higher degree of multifractality in the N-S direction compared with the E-W direction over these scales, especially for the bottomside F-region. As shown by Fig. 6c,f, the origin of multifractality is the small-scale intermittent events in the fat-tail PDFs. These large-amplitude coherent structures embedded in ionospheric intermittent turbulence are driven by amplitude-phase synchronization related to multiscale interactions that lead to large-amplitude energy bursts^33,34^. A view of the multifractal behavior of this event for a time sequence of images before and after the EPD reconnection is given in Supplementary Figs S1 and S2.

**Figure 8:**
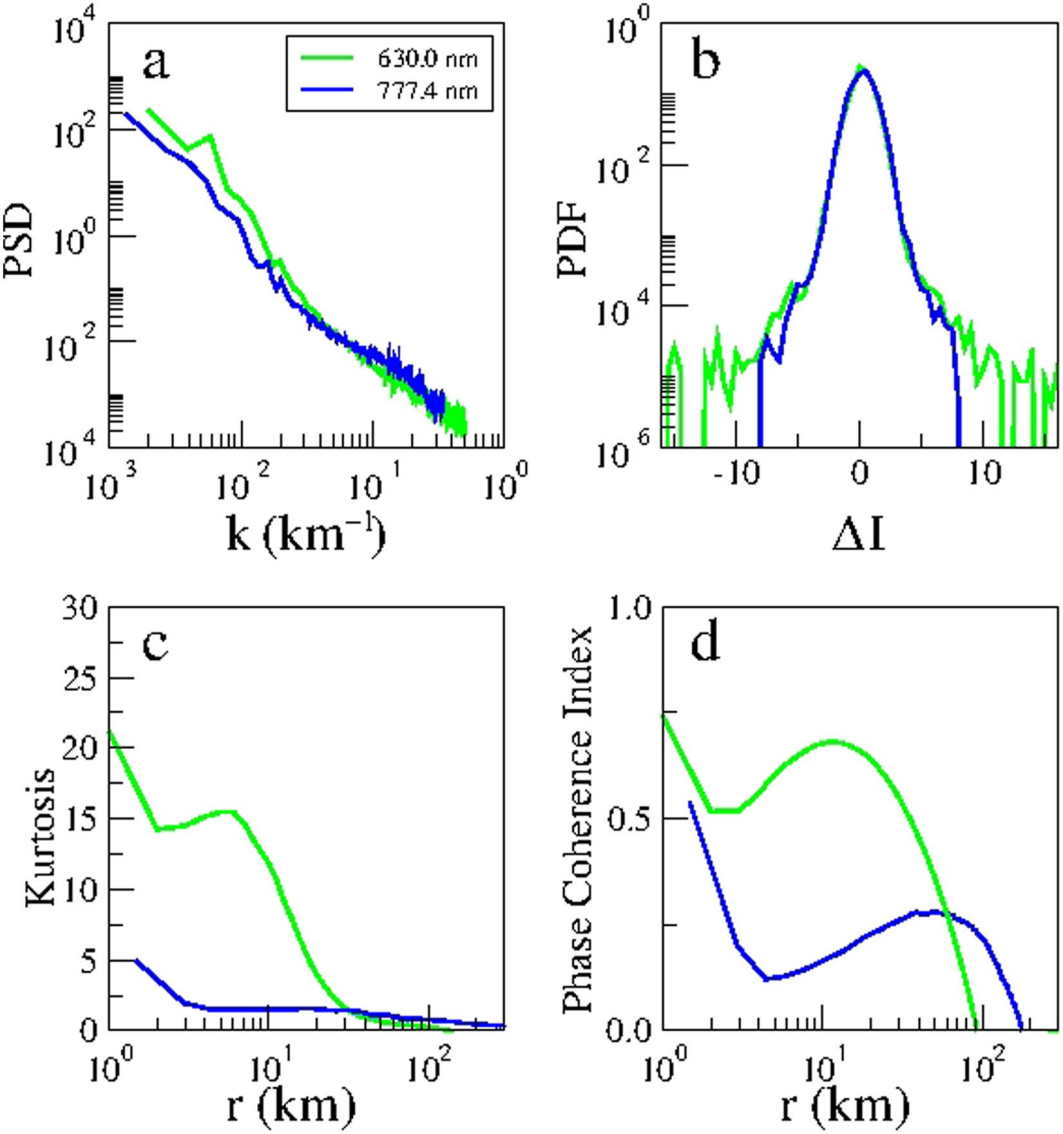
Nonlinear analysis of simultaneous optical imaging of the bottomside (OI 630.0 nm emission) and peak (OI 777.4 nm emission) F-region ionosphere on 28 September 2002: (**a**) Total power spectral density (PSD) as a function of the wavenumber *k*. (**b**) Probability density function (PDF) as a function of the normalized two-point differences of the emissions intensity (Δ*I*) for the spatial scale *r* = 60 km. (**c**) kurtosis and (**d**) phase coherence index as a function of the spatial scale *r* for two-point differences of the emission intensity (*δI*) in the North-South direction of the OI 630.0 nm (21:48:08 LT, green) and OI 777.4 nm (21:46:04 LT, blue) emissions.

Next we apply the aforementioned tools to distinguish the nature of ionospheric turbulence, after the onset of EPD reconnection, using simultaneous multi-spectral optical imaging at two different heights by comparing for the sake of clarity in Fig. 8 the total power spectral density, and the probability density function for the spatial scale 60 km, kurtosis and phase coherent index of the N-S emission intensity of OI 630.0 nm (21:48:08 LT) and OI 777.4 nm (21:46:04 LT) emissions taken from Figs 6 and 7. It follows from Fig. 8 that the power spectral density of the bottomside F-region is steeper than the peak F-region as seen in Fig. 6, and the degree of non-Gaussianity (PDF) and intermittency (kurtosis and phase coherence index) in the bottomside F-region is stronger than the peak F-region, in agreement with previous rocket and satellite studies that showed very different features of ionospheric turbulence in the bottomside and topside F-regions^35–37^. Note that it is not straightforward to make a quantitative comparison of the scaling exponents of Fig. 7c,f because the ionospheric turbulence in the bottomside (peak) F-region is not (is) Kolmogorov-like.

## Nonlinear analysis of ionospheric intermittent turbulence on 9 November 2002

**Figure 9:**
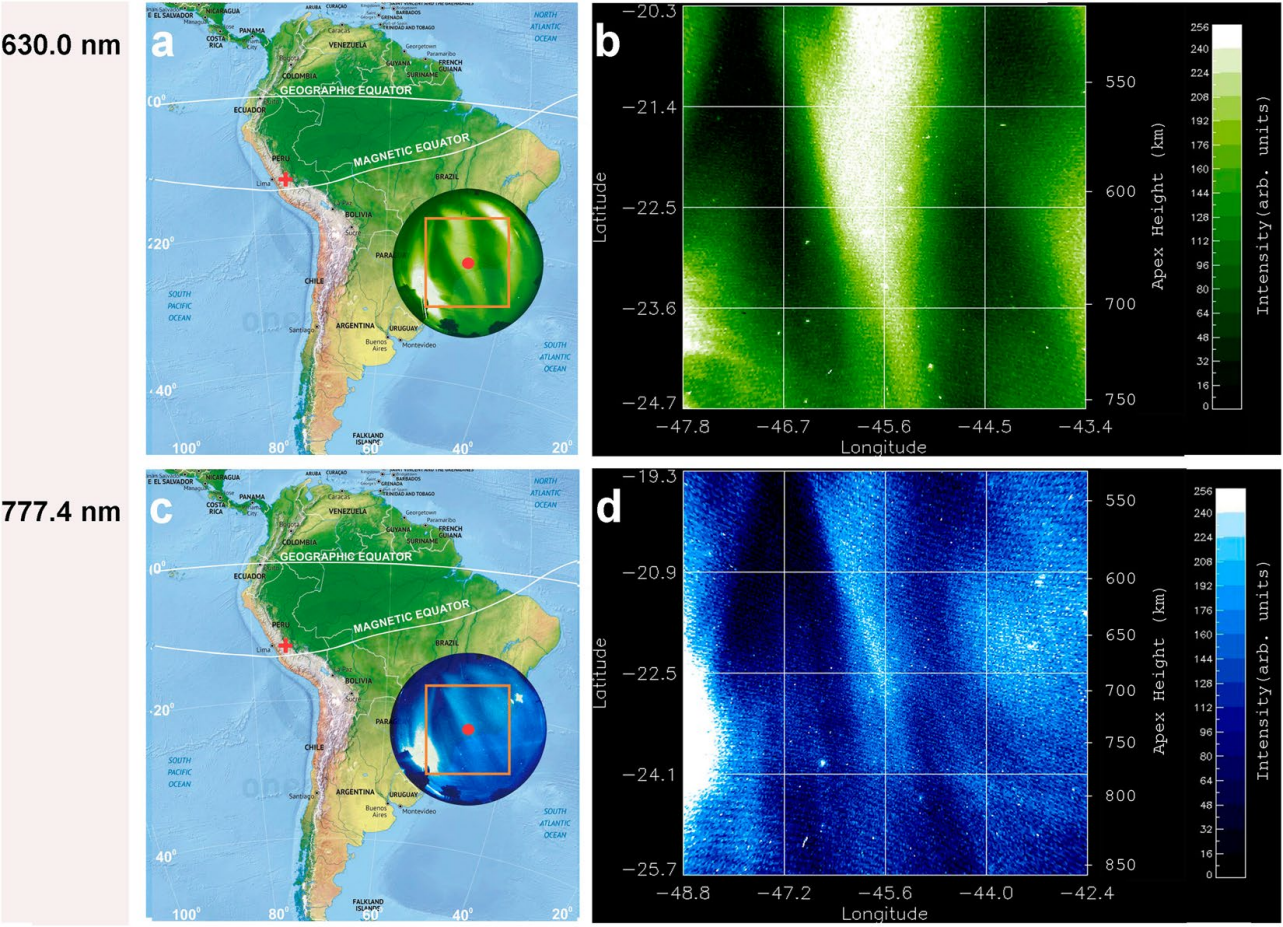
Multi-spectral optical imaging of equatorial plasma depletions on 9 November 2002. Map of the multi-spectral all-sky imaging on 9 November 2002 (similar to Fig. 1). The time of images in Fig. 9a,b is 21:11:57 LT, and in Fig. 9c,d is 21:14:31 LT. (Fig. 9a,c is modified from the Map of South America by onestopmap.com).

**Figure 10:**
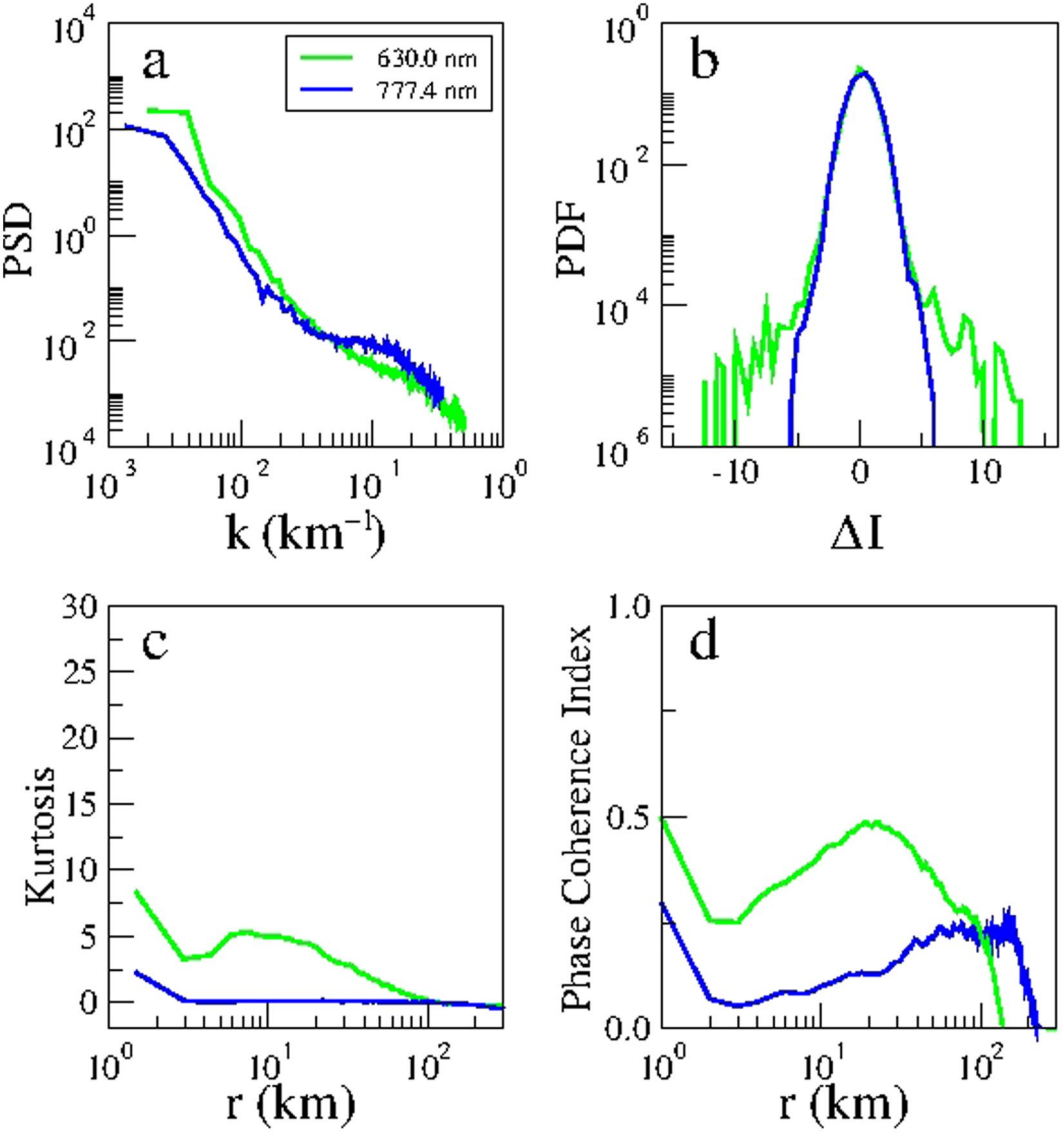
Nonlinear analysis of simultaneous optical imaging of the bottomside (OI 630.0 nm emission) and peak (OI 777.4 nm emission) F-region ionosphere on 9 November 2002: (**a**) Total power spectral density (PSD) as a function of the wavenumber *k*. (**b**) Probability density function (PDF) as a function of the normalized two-point differences of the emission intensity (Δ*I*) for the spatial scale *r* = 60 km. (**c**) kurtosis and (**d**) phase coherence index as a function of the spatial scale *r* for two-point differences of the emission intensity (*δI*) in the N-S direction of the OI 630.0 nm (21:11:57 LT, green) and OI 777.4 nm (21:14:31 LT, blue) emissions.

In order to demonstrate the consistency with our results of 28 September 2002, we carried out nonlinear analysis of multi-spectral images taken by the all-sky imager at the same site and at same times on the night of 9 November 2002. The geomagnetic activity on this day was quiet with the daily planetary index ΣKp of 10+, the daily Ap index of 6, and the daily solar flux index F10.7 of 185.5. Figure 9a,c shows the raw images of OI 630.0 nm (21:11:57 LT) and OI 777.4 nm (21:14:31 LT) emissions, respectively, on 9 November 2002. Figure 9b,d shows the corresponding linearized images covering an area of 500 × 500 km^2^ and 750 × 750 km^2^, respectively. For consistency of statistics, the dimensions of the areas in Fig. 9b,d are the same as Fig. 1b,d. We applied the same nonlinear analysis of Fig. 8 to the images of Fig. 9b,d. Figure 10 shows the total power spectral density and the probability density function for the spatial scale 60 km, and the kurtosis and phase coherence index of the N-S emission intensity of OI 630.0 nm and OI 777.4 nm emissions. Evidently, Fig. 10 shows that the slope of power spectral density in the bottomside F-region ionosphere is steeper than in the peak F-region ionosphere, and the degree of PDF non-Gaussianity and intermittency (kurtosis and phase coherence index) in the bottomside F-region ionosphere is stronger than in the peak F-region ionosphere, which confirms the results of Fig. 8. Hence, our results for 28 September 2002 are robust and in agreement with the results of 9 November 2002. The value of the normalized variance computed by Eq. (1) for the power spectrum of Fig. 10a for OI 630.0 nm (OI 777.4 nm) emission is 0.341 (0.180), very close to the value of 0.342 (0.181) obtained by computing the normalized variance of the emission intensity of the linearized images of Fig. 9b,d.

## Discussion and Conclusions

Coherent structures and intermittent turbulence are well-known in the atmosphere^38^, solar wind^29–31^, and auroral ionosphere^39^. In this paper, we investigated the first optical imaging of intermittent turbulence in the equatorial ionosphere. In the atmosphere, kinematic coherent structures such as vortices (eddies) dominate the atmospheric intermittent turbulence. In the solar wind, magnetic coherent structures such as current sheets and magnetic flux ropes dominate the interplanetary intermittent turbulence. Coherent structures and turbulence in the bottomside and topside F-region ionosphere have been observed previously by rockets, satellites, radars, and numerical simulations. The measured power spectral densities of EPDs display various power-law spectral indices that signal a degree of self-similar scale invariance over a range of scale sizes^35^. In contrast to the well-developed Kolmogorov turbulence in the topside equatorial F-region where an inertial subrange with a spectral slope −5/3 has been observed by satellites^36,37^, the bottomside EPDs do not seem to be the product of Kolmogorov turbulence but rather seem to involve coherent steepened structures such as shocks and solitary waves^35^. This is consistent with the computed power spectral densities of Fig. 6a,d, which show that the spectral index is −2.3 in the bottomside F-region and −1.5 (i.e., close to the Kolmogorov spectral index −5/3) in the peak F-region.

Nonlinear steepened waves such as shocks, solitons and vortices are ubiquitous in turbulent plasmas^31,34,40^. Nonlinear coherent structures such as shocks and solitary waves, resulting from nonlinear steepening of waveforms, in the equatorial ionosphere have been studied by a number of papers. McClure and Hanson^41^ reported the detection by the OGO-6 satellite over Brazil of large-amplitude density irregularities related to EPDs with amplitudes comparable to the largest auroral density irregularities. A theoretical analysis of Chaturvedi and Ossakow^42^ shows that nonlinear evolution of the collisional Rayleigh-Taylor instability in the equatorial ionosphere can evolve to steepened structures with a power-law spectrum. Costa and Kelley^43^ showed that the Rayleigh-Taylor instability that initiates in the bottomside equatorial F-region can nonlinearly develop very sharp gradients leading to the formation of steepened structures responsible for the power-law spectra observed by a rocket experiment in Natal, Brazil. Shock waves were observed by numerical simulation^44^ of the generalized Rayleigh-Taylor instability at the bottomside and topside F-region equatorial ionosphere, confirmed by rocket and satellite *in situ* data^45^. Hysell *et al*.^46,47^ proposed a model of plasma steepening, evolving from plasma advection that occurs on the vertical leading edges of plasma depletion wedges, to interpret shock waves detected in the equatorial ionosphere by rockets launched from Kwajalein Atoll. Jahn and Labelle^48^ measured shocklike structures characterized by the density waveforms at the bottomside and topside F-region of the equatorial ionosphere in a rocket experiment in Alcântara, Brazil. Hysell and Kelley^49^ and Hysell^35^ obtained analytical and numerical solutions of solitary waves for a one-dimensional model of steepened structures which bear a striking resemblance to kilometer-scale waves associated with EPDs observed by the AE-E satellite in the bottomside layers of the equatorial ionosphere. At intermediate-scale sizes, multiscale interactions are acting as energy is transferred from the vertical steepened structures to the horizontal kilometer-scale waves to small-scale waves and then diffusively dissipated^35^. Berthelier *et al*.^50^ reported observations of equatorial plasma waves that indicate the excitation of lower-hybrid solitary structures induced by lightning and the simultaneous occurrence of ion heating in large-scale EPDs that form at night during disturbed geomagnetic conditions. Numerical simulations of Huba and Joyce^11^ showed that during the bifurcation process of EPDs, small-scale structures can interact and merge to form large-scale structures. In addition, numerical simulations of Yokoyama *et al*.^12^ showed that bifurcation, pinching and merging can lead to the formation of turbulent density structures inside EPDs. The steeper slope of power spectral density, the thicker fat-tail in the probability density function, and the higher degrees of kurtosis and phase coherent indices of the bottomside F-region compared to the peak F-region, seen in Figs 8 and 10, are the manifestations of aforementioned nonlinear coherent structures in the bottomside F-region equatorial ionosphere.

The morphology of the airglow images in Figs 1–5 shows that the EPDs are highly anisotropic. In fact, the decomposition of nonlinear analyses into N-S and E-W directions confirms that the degree of complexity (intermittency, non-Gaussianity, multifractality) at these scales is stronger in the N-S direction compared to the E-W direction, especially at the bottomside F-region. Note, however, that the airglow imagery is ambiguous, and the N-S variations conflate meridional and apex-height variations. Nevertheless, when we find that N-S variations have a longer characteristic scale it implies that both meridional and apex-height variations have a longer scale than longitudinal (E-W) variations. Furthermore, airglow is a representation of plasma density passed through a complicated instrument function that acts like an averaging kernel. Some of the structures we see are an artefact of the imaging process and would not be present, for example, in radar images or *in-situ* rocket and satellite measurements. Finally, whereas magnetic field lines are equipotentials, they are not isodensity curves, and the field-aligned mapping of EPDs is very imperfect. Hence, it is difficult to relate airglow intensity to plasma state variables and therefore difficult to draw precise quantitative conclusions.

In conclusion, we reported multi-spectral all-sky observations of equatorial plasma depletions in Brazópolis, Brazil, on the nights of 28 September 2002 and 9 November 2002. Our optical observations at two different altitudes at same times provide new insights on the nonlinear dynamics of EPDs. So far the studies of EPD turbulence were mostly based on analyses of power spectra and waveforms, without quantifying the degree of intermittency or multifractality which are essential for understanding the EPD dynamics and structures. By applying the nonlinear techniques of kurtosis and phase-coherence index, we quantified the degree of amplitude-phase synchronization as a function of spatial scale which demonstrated that the amplitude-phase synchronization due to multiscale interactions is responsible for generating coherent structures and intermittent turbulence in the equatorial ionosphere. By computing the scaling exponents for higher moments of the emission intensity, we quantified the degree of multifractality and proved the multifractal nature of equatorial plasma depletions. Our new nonlinear tools showed that the degree of intermittency and non-Gaussianity at the bottomside F-region ionosphere is higher than at the peak F-region ionosphere, due to a higher rate of occurrence of nonlinear coherent structures such as steepened kilometer-scale waves. Our results are consistent for observations on two different dates. In particular, we showed for the first time that optical imaging using all-sky imagers can be readily used to characterize the nonlinear spatiotemporal dynamics of equatorial plasma depletions, which can be used in conjunction with radars, rockets, satellites and numerical simulations for future studies of the equatorial ionosphere turbulence.

## Electronic supplementary material

Supplementary Information

Video OI630.0 nm

Video OI777.4 nm
