# Supplementary material for: Multi-spectral optical imaging of the spatiotemporal dynamics of ionospheric intermittent turbulence
Source: Sci Rep. 2018 Jul 12;8:10568. doi: 10.1038/s41598-018-28780-5 (PMC6043611; doi:10.1038/s41598-018-28780-5)
Supplement: Supplementary file 1 — Supplementary Information [file 41598_2018_28780_MOESM1_ESM.pdf]

Multi-spectral optical imaging of the spatiotemporal dynamics of ionospheric intermittent turbulence
Supplementary Information

Abraham C.-L. Chian, José R. Abalde, Rodrigo A. Miranda, Felix A. Borotto, David L. Hysell, Erico L. Rempel and David Ruffolo

An overview of optical imaging of the OI 630.0 nm (777.4 nm) emissions, for the first double EPD reconnection observed in the South Atlantic Magnetic Anomaly on 28 September 2002, is given in Figs. S1 and S2 showing a time sequence of three images for each emission that complements Figs. 4 and 5, respectively. To facilitate visualization of a broader picture of the complex EPD interactions, each image in Fig. 4 (5) covers an area of 1500x1500 km^2 (1530x1530 km^2). In contrast, each image in Fig. S1 (S2) covers an area of 500x500 km^2 (750x750 km^2), the same as Fig. 1b (1d). This choice of smaller image area allows us to perform a better quantitative analysis. Note that the timing of the images in Figs. S1 and S2 are not all the same as Figs. 4 and 5. Like Fig. 4, the images in Fig. S1 exhibit the complex EPD interactions seen in Table 1. In Fig. S1 we identify in detail the first EPD reconnection event, including the dynamical phenomena of EPD connection, disconnection and reconnection by following the temporal evolution of three EPD structures: A, B and C. In Fig. S1a (S1d), A, B and C are connected at the two bifurcation sites indicated by two arrows. In Fig. S1b (S1e), C is disconnected from B at the disconnection site indicated by an arrow, while A remains connected to B. In Fig. S1c (S1f), C is reconnected to A, at the reconnection site indicated by an arrow, to form a merged elongated structure which is also connected to B. It follows from Figs. S1 and 4 and Table 1 that the dynamics of EPD structures are the same at the two different altitudes at same times. In Fig. S2 we identify in detail the second EPD reconnection event by following the temporal evolution of three EPD structures: X, Y and Z. In Fig. S2a (S2d), Y and Z are connected forming an elongated EPD structure Y-Z, but X is not connected to Y-Z within the field-of-view of the all-sky imager; the arrow marks the site of an EPD bifurcation. In Fig. S2b (S2e), Z is disconnected from Y at the disconnection site marked by an arrow. In Fig. S2c (S2f), X is reconnected to Z at the reconnection site marked by an arrow to form an elongated EPD structure X-Z.

Next we study the spatiotemporal variation of the multifractal characteristics of the equatorial ionosphere turbulence on 28 September 2002, by using the time sequence of images in Figs. S1 and S2 to compute the scaling exponent $\zeta(p)$ of the p-th order structure function for two-point differences of the emission intensity (δI) in the N-S direction of the OI 630.0 nm (OI 777.4 nm) emissions at three instances prior to (1, 2) and after (3) the EPD reconnection: (1) A-B-C connected as seen in Fig. S1a (S1d), Y-Z connected as seen in Figs. S2a (S2d); (2) C disconnected from A-B as seen in Fig. S1b (S1e), Z disconnected from Y as seen in Fig. S2b (S2e); and (3) A-B-C reconnected as seen in Fig. S1c (S1f), X-Z reconnected as seen in Fig. S2(c) (S2f). Note that

the timing of Figs. S1c (S1f) and S2c (S2f) is the same as Figs. 1b (1d), 4c (4f), and 5c (5f). The results are plotted in Figure S3 which shows that the scaling exponent is capable of quantifying the degree of multifractality of nonlinear spatiotemporal dynamics of equatorial plasma depletions.

Figure S1 - Supplementary

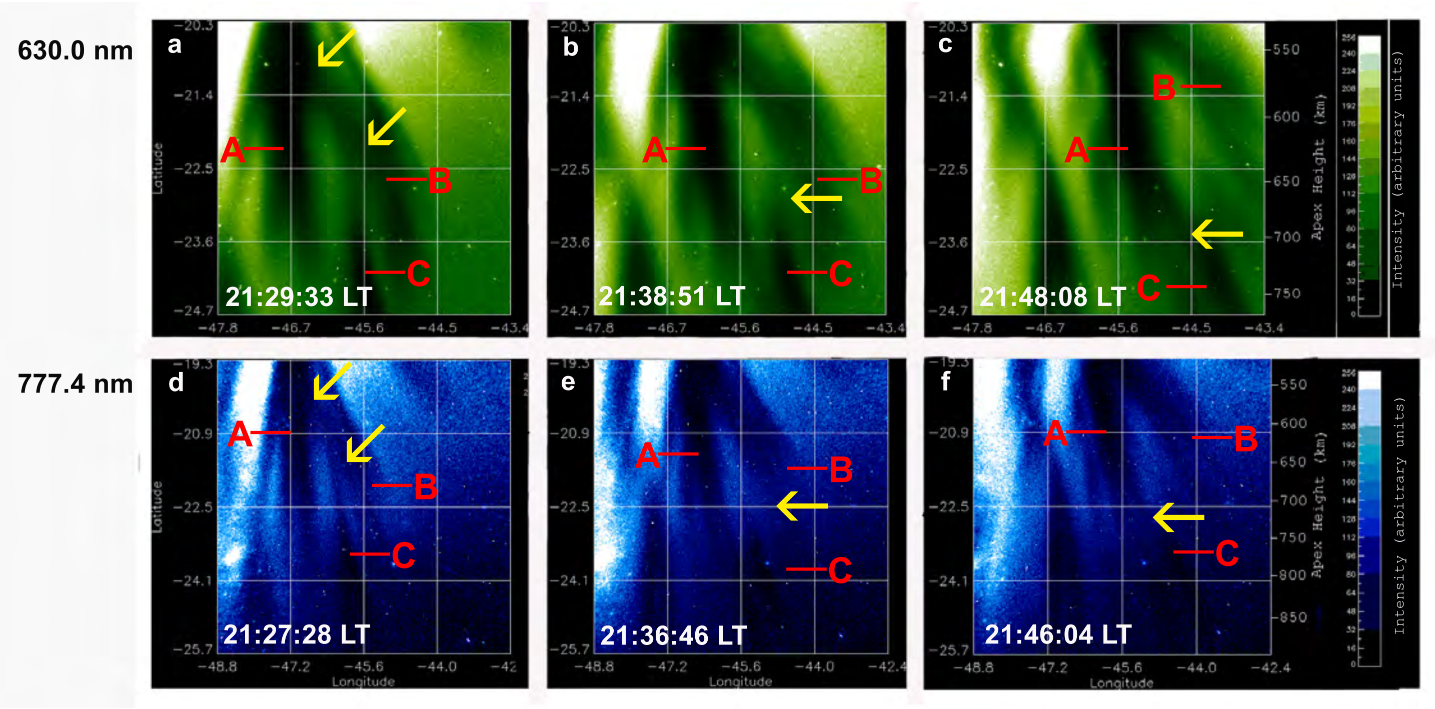

Figure S1: Time sequence of linearized images of the OI 630.0 nm and OI 777.4 nm emissions of the first EPD reconnection event of 28 September 2002. (a, d): A-B-C connected, the two arrows indicate the sites of bifurcation and connection of A and B-C and of B and C. (b, e): C disconnected from A-B, the arrow indicates the site of disconnection. (c, f): A-B-C reconnected, the arrow indicates the site of reconnection.

Figure S2 - Supplementary

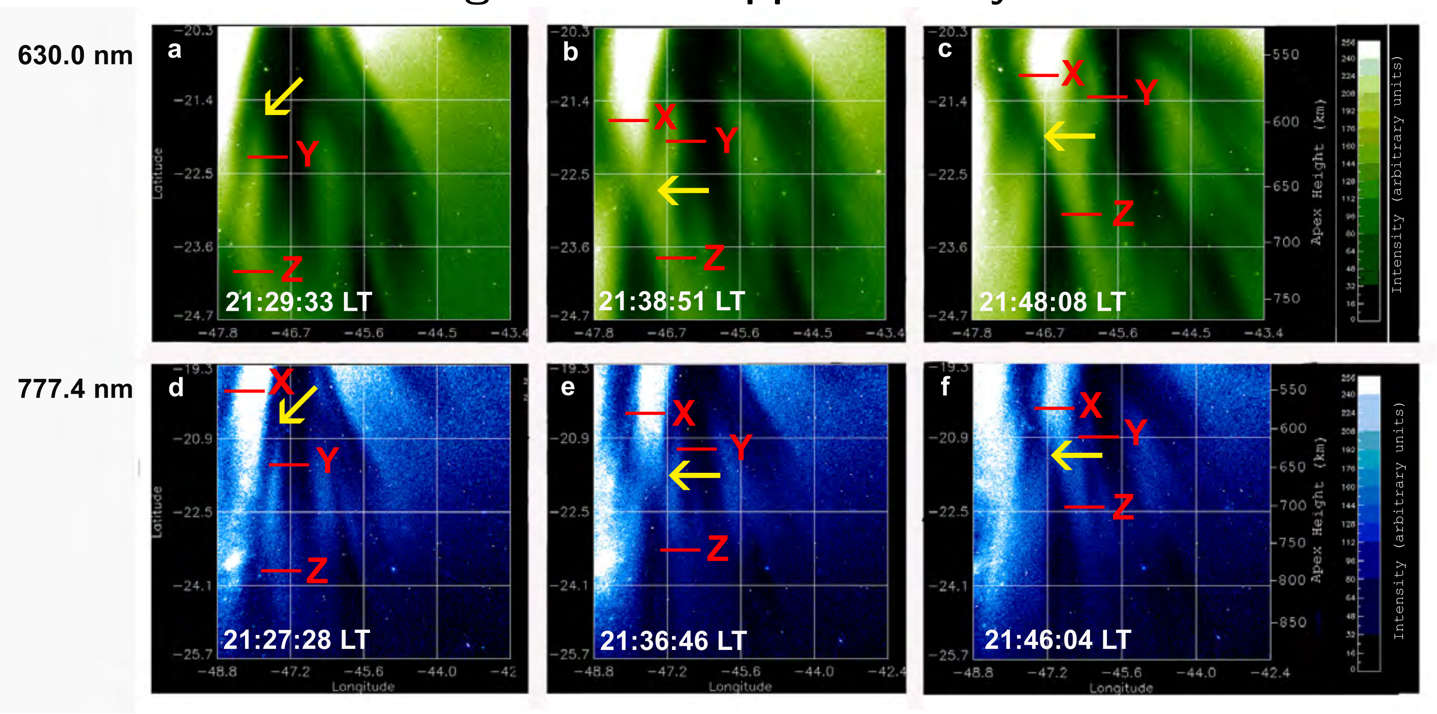

Figure S2: Time sequence of linearized images of the OI 630.0 nm and OI 777.4 nm emissions of the second EPD reconnection event of 28 September 2002. (a, d): Y-Z connected, the arrow indicates a site of bifurcation; X is out of the field of view in Fig. S2a. (b, e): Z disconnected from Y, the arrow indicates the site of disconnection. (c, f): X-Z reconnected, the arrow indicates the site of reconnection.

Figure S3 - Supplementary

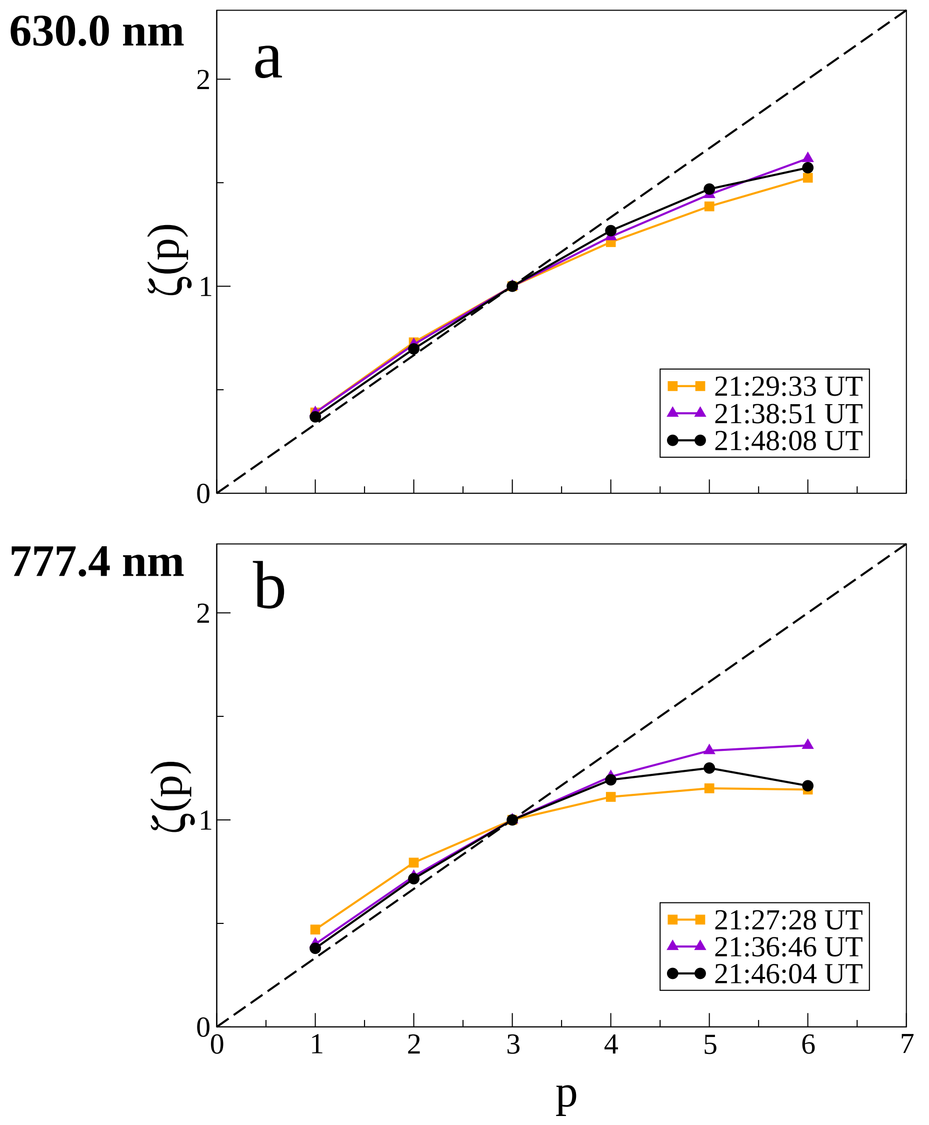

Figure S3. Multifractal spatiotemporal dynamics of ionospheric intermittent turbulence. Scaling exponent $\zeta(p)$ of the p-th order structure function for two-point differences of the emission intensity (δI) in the N-S direction of the: (a) OI 630.0 nm and (b) OI 777.4 nm emissions at different times for the linearized images of Figs. S1 and S2. The Kolmogorov K41 self-similar scaling is indicated by a black dashed line.

Videos for event of 28 September 2002 - Supplementary

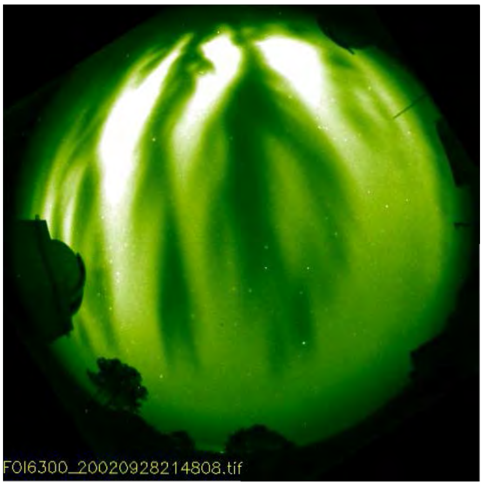

Video S1 - OI630.0 nm
Click on picture to play

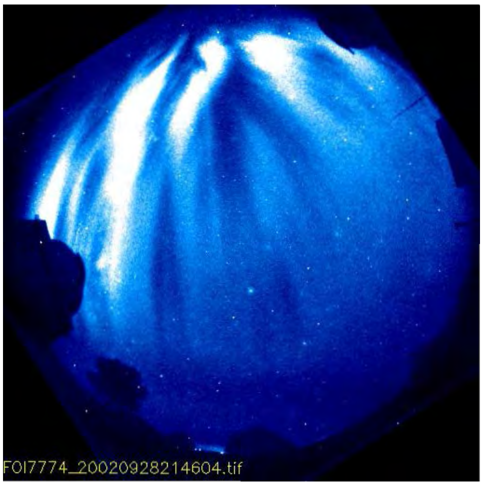

Video S2 - OI777.4 nm
Click on picture to play

Videos of all-sky airglow images. These two videos show a time sequence of digital all-sky airglow images obtained in Brazópolis, Brazil (22.5^0 S, 45.6^0 W; dip latitude 17.5^0 S; altitude 1860 m) on the night of 28 September 2002. The false green (blue) colour of OI 630.0 nm (OI 777.4 nm) images correspond to a radius of 1900 km (2070 km) at a reference height of 280 km (330 km) centered over the imager zenith as presented in 512 x 512 pixels, rendering a spatial resolution of about 2x2 km^2 per pixel. Top and right corresponds to the north and east, respectively. The southwest and northeast corners of the images show the buildings and trees surrounding the National Laboratory of Astrophysics (LNA) which reduce the apparent field of view. However, they do not affect our analyses in any way. The present airglow data did not suffer any interference from the moonlight. The series of images in this video clearly shows an eastward propagation of the complex event of equatorial plasma depletions in the successive images. The period of observation of the OI 630.0 nm emission was from 19:48:41 to 00:12:12 LT and the sampling period of the images is approximately 5 min. The first video shows 61 airglow images with a submission rate of 1 picture/s. The period of observation of the OI 777.4 nm emission was from 20:08:28 to 00:10:07 LT and the sampling period of the images is approximately 5 min. The second video shows 53 airglow images with a submission rate of 1 picture/s.
